# *Trichophyton indotineae* Infection, São Paulo, Brazil, 2024

**DOI:** 10.3201/eid3105.250048

**Published:** 2025-05

**Authors:** João Nobrega de Almeida, Amanda R. dos Santos, Mario Roberto de S. Trindade, Jeremy A. W. Gold, Fernanda P.M. Razo, Sarah S. Gonçalves, Erick G. Dorlass, Renato de Mello Ruiz, Jacyr Pasternak, Cristovão L.P. Mangueira, Silke Uhrlaß, Pietro Nenoff, Shyam B. Verma, André M. Doi, Marines D.V. Martino

**Affiliations:** Federal University of São Paulo, São Paulo, Brazil (J. Nobrega de Almeida Jr); Antimicrobial Resistance Institute of São Paulo, São Paulo (J. Nobrega de Almeida Jr); Hospital Israelita Albert Einstein, São Paulo (J. Nobrega de Almeida Jr, M.R. de S. Trindade, E.G. Dorlass, R. de Mello Ruiz, J. Pasternak, C.L.P. Mangueira, A.M. Doi, M.D.V. Martino); Centers for Disease Control and Prevention, Atlanta, Georgia, USA (A.R. dos Santos, J.A.W. Gold); Policlínica Lincoln de Freitas Filho, Rio de Janeiro, Brazil (F.P.M. Razo); Federal University of Espírito Santo, Vitoria, Brazil (S.S. Gonçalves); Labopart Medical Laboratories, Roetha OT Moelbis, Germany (S. Uhrlaß, P. Nenoff); Nirvan and In Skin Clinics, Vadodara, Gujarat, India (S.B. Verma)

**Keywords:** *Trichophyton indotineae*, fungi, drug resistance, fungal, tinea, whole-genome sequencing, Brazil

## Abstract

We report an extensive, terbinafine-resistant (squalene epoxidase F397L mutation) *Trichophyton indotineae* infection in a previously healthy businessman from São Paulo, Brazil. The patient had previously traveled to France, Spain, and the United States. Clinician awareness, laboratory testing capacity, and surveillance are essential to prevent *T. indotineae* spread and inform healthcare practices.

*Trichophyton indotineae* is an anthropophilic, frequently terbinafine-resistant fungus causing recalcitrant dermatophytosis. It has become endemic in South Asia; cases are documented across 6 continents, and possible local US transmission has been reported (*1*,*2*). São Paulo, Brazil, South America’s largest city, is known for its global business connections and frequent international travelers.

In September 2024, a previously healthy São Paulo man in his 40s sought treatment for difficult-to-treat tinea cruris. In October 2023, he traveled to Paris and Barcelona, and 30 days later, he traveled to Boston, Massachusetts, USA. He had not traveled to Asia. Six weeks after he returned home, he noticed pruritic, erythematous, bilateral groin lesions. He treated the lesions with topical betamethasone and ketoconazole, but they worsened. After consulting multiple dermatologists, he was prescribed oral terbinafine (9 weeks), with no improvement.

Subsequently, another dermatologist, accessed by telemedicine, performed a full-body examination and identified additional lesions at the dorsal region of the left foot. Groin lesions had irregular borders, erythematous inflammatory areas, and reddish scaly plaques (Figure 1); the dorsal region of the left foot had small scaly erythematous plaques. Direct microscopy from groin and foot skin scrapings was positive, and a culture of groin scrapings showed dermatophyte mold.

**Figure 1:**
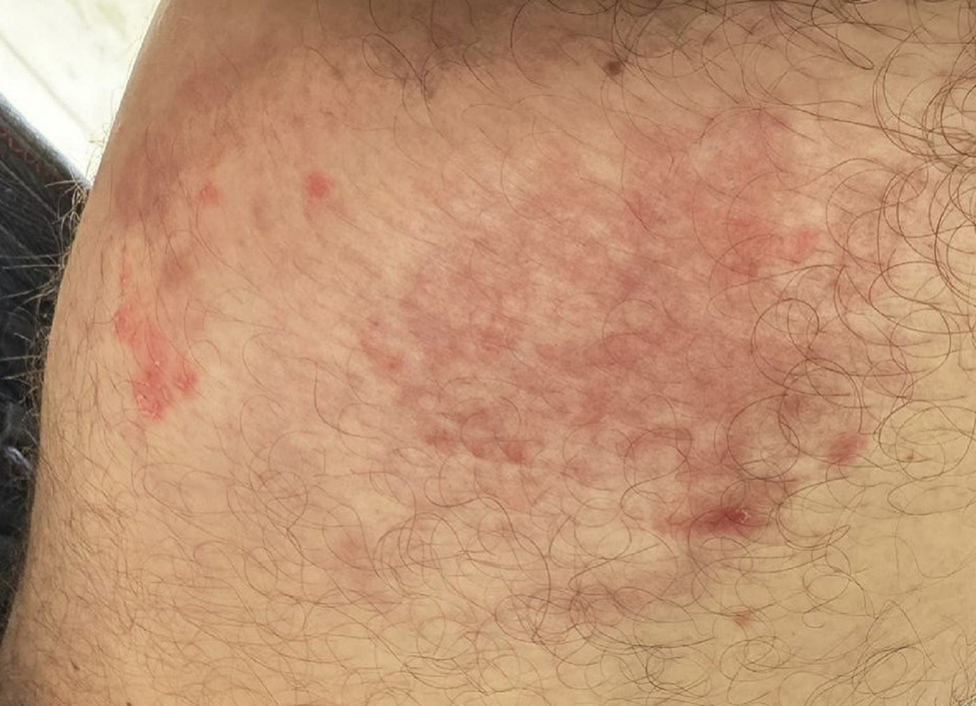
Atypical tinea cruris from *Trichophyton indotineae* infection, São Paulo, Brazil, 2024. A photograph of the left groin (provided by the patient) shows lesions characterized by poorly defined margins, hyperemic scaly plaques in the medial region, and an inflammatory infiltrate in the central-lateral area.

Initial matrix-assisted laser desorption/ionization time-of-flight mass spectrometry analysis using the Biotyper 3.0 database (Bruker Daltonics, https://www.bruker.com) identified the organism as part of the *Trichophyton mentagrophytes* group. Additional analysis using the MSI-2 database (Université Paris VI, https://msi.happy-dev.fr) identified *T. indotineae* with high confidence (*3*). The physician switched the patient’s treatment to itraconazole (200 mg/d), with substantial improvement noted after 8 weeks.

We confirmed the identification of *T. indotineae* by using internal transcribed spacer ribosomal DNA sequencing analysis (GenBank accession no. PQ726960). Antifungal susceptibility testing (Appendix) for the isolate using broth microdilution showed high MICs against terbinafine (>4 mg/L) and fluconazole (32 mg/L) and strong in vitro activity against itraconazole (0.016 mg/L) and voriconazole (0.125 mg/L) (*4*). Currently, clinical breakpoints for interpreting antifungal susceptibility testing of *T. indotineae* do not exist.

We performed genomic analyses to assess the isolate’s possible origins and to detect the presence of squalene epoxidase (*SQLE*) gene mutations associated with terbinafine resistance (*5*,*6*). We performed whole-genome sequencing by using the NextSeq 550 system (Illumina, https://www.illumina.com). We deposited read data into National Center for Biotechnology Institute Sequence Read Archive database (https://www.ncbi.nlm.nih.gov/sra; BioProject no. PRJNA1196410). We downloaded an additional 347 *T. indotineae* sequences from isolates collected in 14 countries from the Sequence Read Archive database (Appendix Table 2) and included them in the genomic analysis (A.R. dos Santos et al., unpub. data). We performed single-nucleotide polymorphism (SNP) identification and phylogenetic analysis using MycoSNP version 1.5 (https://github.com/CDCgov/mycosnp-nf), with *T. indotineae* strain TIMM20114 as the reference genome. We performed SNP identification in the *SQLE* gene by mapping filtered reads to a reference sequence (OM313310.1) by using the Burrows-Wheeler Aligner (https://bio-bwa.sourceforge.net), followed by variant calling with freebayes (https://github.com/freebayes/freebayes). Genomic analysis showed that the isolate from the patient from Brazil was closely related to other terbinafine-resistant *T. indotineae* isolates from 13 countries (Figure 2). Among isolates analyzed, the isolate from the patient from Brazil was most genetically similar to one from Lower Saxony, Germany (21 SNPs distance). The isolate was collected from a patient with terbinafine-resistant *T. indotineae* in June 2022, and, like the isolate from the patient in Brazil, it had a terbinafine resistance–conferring *SQLE* gene mutation (F397L).

**Figure 2:**
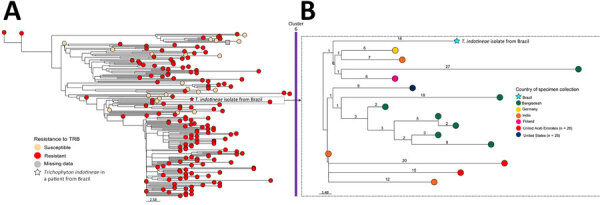
*Trichophyton indotineae* phylogenetic tree analysis by terbinafine susceptibility profile and country of origin for *T. indotineae* infection, São Paulo, Brazil, 2024. A) Neighbor-joining phylogenetic tree, which includes terbinafine-resistant and -susceptible *T. indotineae* isolates belonging to cluster 6. Isolates were considered terbinafine resistant if they had a missense point mutation in the squalene epoxidase (*SQLE*) gene for *Trichophyton* spp. linked to terbinafine resistance (*5*,*6*). B) Subcluster containing the isolate from the patient in Brazil. Among the 12 isolates in that subcluster, 5 resistant isolates, including an isolate from Germany, had the *SQLE* substitution F397L. Branch lengths represent the single-nucleotide polymorphism distance between isolates, and leaf colors correspond to the different countries in which each specimen was collected. The neighbor-joining tree and map were visualized together with each sample’s metadata using Microreact version 252 (https://docs.microreact.org). Scale bar represents number of single-nucleotide polymorphisms. TRB, terbinafine.

We report a case of tinea caused by terbinafine-resistant *T. indotineae* in a businessman from Brazil who traveled to Europe and the United States. Genomic analysis revealed that the patient’s isolate contained a terbinafine resistance–conferring *SQLE* mutation, F397L, and fit within a predominantly terbinafine-resistant cluster of isolates collected from countries across North America, Europe, and Asia. Although it is uncertain where the patient acquired infection, his isolate’s close relatedness to one from Germany suggests possible acquisition in Europe. However, additional analysis, including of isolates from Barcelona, Paris, and Boston, is essential to confirm where the infection was acquired.

Clinicians should be vigilant for possible *T. indotineae* infection in persons who have traveled abroad or seek treatment for difficult-to-treat tinea because local transmission may occur. Clinicians should advise those patients about strategies to prevent transmission (*7*), need for prolonged therapy (e.g., >6 weeks) with itraconazole (*8*), and importance of avoiding topical corticosteroids and antifungal corticosteroid products, which can worsen tinea infections (*8*).

In Brazil and other resource-limited settings, lack of specialized and well-equipped microbiology laboratories could enable unrecognized introduction and local spread (*9*). Increasing laboratory capacity for dermatophyte species identification, antifungal susceptibility testing, and genomic epidemiology studies is essential for monitoring transmission patterns and guiding effective treatment strategies (*10*). Combining matrix-assisted laser desorption/ionization time-of-flight mass spectrometry with the improved MSI-2 database might help overcome challenges of identifying *T. indotineae* in skin scraping cultures in clinical laboratories. That approach might also enhance epidemiologic understanding of global spread of that species and contribute to improved patient care (*3*). In conclusion, this case highlights the importance of integrating clinical, microbiological, and genomic data to address spread of antimicrobial-resistant pathogens in an increasingly interconnected world.

Appendix Additional information for *Trichophyton indotineae* infection, São Paulo, Brazil, 2024
