# Supplementary material for: Trichophyton indotineae Infection, São Paulo, Brazil, 2024
Source: Emerg Infect Dis. 2025 May;31(5):1049–51. doi: 10.3201/eid3105.250048 (PMC12044233; doi:10.3201/eid3105.250048)
Supplement: Appendix — Additional information for Trichophyton indotineae infection, São Paulo, Brazil, 2024 [file 25-0048-Techapp-s1.pdf]

Article DOI: https://doi.org/10.3201/eid3105.250048

EID cannot ensure accessibility for supplementary materials supplied by authors. Readers who have difficulty accessing supplementary content should contact the authors for assistance.

Trichophyton indotineae Infection, São Paulo, Brazil, 2024

Appendix

Supplemental Methods

Morphologic Characterization

Morphologic characterization was conducted following the methods outlined by De Hoog et al. (*1*). The isolate was cultivated on Lacrimel agar, which consists of 7 g skimmed milk powder, 10 g honey, 17 g cornmeal agar (Oxoid), 0.05 g chloramphenicol, and 1,000 mL distilled water. The culture was incubated at 25°C for 14 days. After incubation, the colony diameter and descriptions were recorded. Microscopic features were examined by using lactic acid mounts and visualized with an optical microscope at ×40 magnification.

In Vitro Antifungal Susceptibility Testing

Antifungal susceptibility testing was conducted by using the EUCAST E.Def 11.0 broth dilution method. The isolate was evaluated against several antifungal agents, including terbinafine, fluconazole, voriconazole, and itraconazole (all sourced from Sigma-Aldrich in India). *Trichophyton indotineae* was subcultured on potato dextrose agar plates containing cycloheximide (300 mg/L) and chloramphenicol (50 mg/L) and incubated at 25°C for 7 days. A conidia inoculum was prepared in sterile water containing 0.1% Tween-20 by covering the colonies with water and scraping them with a sterile swab. The microconidia were transferred to a sterile tube with 5 mL sterile water and Tween-20 solution and filtered through an 11-µm filter. The suspension was adjusted to a concentration of 2×10^6 to 5×10^6 microconidia/mL, equivalent to McFarland 0.5, as confirmed by using a hemocytometer. This was further diluted 1:10 to achieve a final inoculum concentration of 2×10^5–5 × 105/mL, supplemented with 600 mg/L cycloheximide and 100 mg/L chloramphenicol. After inoculating the *T. indotineae* strain into a tissue-treated microdilution plate containing the antifungal agent to be evaluated, the plate

was incubated at 25°C for 5 days. Using a spectrophotometer, the MIC was visually determined at 50% inhibition of fungal growth. *Aspergillus flavus* ATCC 204304 reference strains were used as controls and assessed after 48 hours of incubation at the same temperature. The isolate was classified as either wild type or non–wild type on the basis of the European Committee on Antimicrobial Susceptibility Testing (EUCAST) epidemiologic cutoff values (ECOFFs) for terbinafine, itraconazole, and voriconazole. It should be noted that ECOFFs for fluconazole have not yet been established.

Supplemental Results

The *T. indotineae* isolate exhibited colonies that grew to a diameter of 5–6 cm, appearing flat with a suede-like texture and ranging in color from white to cream (Appendix Figure, panel C). The reverse sides of the colonies were yellowish to brownish (Appendix Figure, panel D). Microscopic examination revealed the presence of septate, hyaline, and spiral hyphae (Appendix Figure, panels A, B). Additionally, the macroconidia were fusiform to clavate and had thin walls (Appendix Figure, panel A), whereas the microconidia, alongside undifferentiated hyphae, were spherical to pyriform in shape (Appendix Figure, panels A, B).

This *T. indotineae* isolate exhibited low MICs for itraconazole and voriconazole at 0.016 mg/L and 0.125 mg/L, respectively. In contrast, fluconazole showed MIC of 32 mg/L. At the same time, terbinafine had a high MIC of 4 mg/L, categorized as non–wild type (Appendix Table 1).

Reference

1. de Hoog GS, Guarro J, Gené J, Ahmed SA, Al-Hatmi AMS, Figueras MJ, et al. Atlas of clinical fungi, 4th ed. Utrecht (the Netherlands): Westerdijk Institute; 2019.

Appendix Table 1. Antifungal susceptibility testing of *Trichophyton indotineae* isolate*

Antifungal	MIC, mg/L	WT/NWT	ECOFF†
Terbinafine	4	NWT	0.125
Fluconazole	32	ND	ND
Itraconazole	0.016	WT	0.25
Voriconazole	0.125	NWT	1

*ECOFF, epidemiologic cutoff values; ND, not determined; NWT, non–wild type; WT, wild type.
†European Committee on Antimicrobial Susceptibility Testing (EUCAST) E.Def 7.4, E.Def 9.4 and E.Def 11.0 procedures. Version 4.0, 2023,

Appendix Table 2. Characteristics of whole-genome sequences of *Trichophyton indotineae* isolates from human cases downloaded from the National Center for Biotechnology Information

Sample accession no.	Strain	Year of isolation	Geographic origin
SRR31646026	B18430	2017	India
SRR31646025	B18431	2017	India
SRR31645788	B18432	2017	India
SRR31645931	B18433	2017	India
SRR31645898	B18434	2017	India
SRR31645855	B18435	2017	India
SRR31645844	B18436	2017	India
SRR31645833	B18437	2017	India
SRR31645822	B18438	2017	India
SRR31645811	B18439	2017	India
SRR31646024	B18440	2017	India
SRR31646013	B18442	2018	India
SRR31646002	B18443	2018	India
SRR31645991	B18444	2018	India
SRR31645980	B18445	2018	India
SRR31645969	B18447	2019	India
SRR31645894	B18448	2019	India
SRR31645883	B19651	2017	India
SRR31645872	B19652	2017	India
SRR31645799	B19653	2017	India
SRR31645787	B19658	2017	India
SRR31645776	B19659	2017	India
SRR31645765	B19660	2017	India
SRR31645754	B19663	2017	India
SRR31645743	B19664	2017	India
SRR31645732	B19665	2017	India
SRR31645721	B19666	2017	India
SRR31645710	B19669	2017	India
SRR31645953	B19670	2017	India
SRR31645942	B19671	2017	India
SRR31645930	B19672	2017	India
SRR31645697	B19673	2017	India
SRR31646040	B19674	2017	India
SRR31646029	B19675	2017	India
SRR31645918	B19676	2017	India
SRR31645907	B19677	2017	India
SRR31645902	B19678	2017	India
SRR31645901	B19679	2017	India
SRR31645900	B19680	2017	India
SRR31645899	B19681	2017	India
SRR31645897	B19682	2017	India
SRR31645896	B19683	2017	India
SRR31645895	B19684	2017	India
SRR31645862	B19685	2017	India
SRR31645861	B19686	2017	India
SRR31645860	B19687	2017	India
SRR31645859	B19688	2017	India
SRR31645858	B19689	2017	India
SRR31645857	B19690	2017	India
SRR31645856	B19691	2017	India
SRR31645854	B19692	2017	India
SRR31645853	B19693	2017	India
SRR31645852	B19694	2017	India
SRR31645851	B19695	2017	India
SRR31645850	B19696	2017	India
SRR31645849	B19697	2017	India
SRR31645848	B19698	2017	India
SRR31645847	B19701	2017	India
SRR31645846	B19702	2017	India
SRR31645845	B19703	2017	India
SRR31645843	B19704	2017	India
SRR31645842	B19705	2017	India
SRR31645841	B19706	2017	India
SRR31645840	B19707	2017	India
SRR31645839	B19708	2017	India
SRR31645838	B19709	2017	India
SRR31645837	B19710	2017	India

Sample accession no.	Strain	Year of isolation	Geographic origin
SRR31645836	B19711	2017	India
SRR31645835	B19712	2017	India
SRR31645834	B19714	2017	India
SRR31645832	B19715	2017	India
SRR31645831	B19716	2017	India
SRR31645830	B19717	2017	India
SRR31645829	B19718	2018	India
SRR31645828	B19719	2018	India
SRR31645827	B19720	2018	India
SRR31645826	B19721	2018	India
SRR31645825	B19722	2018	India
SRR31645824	B19723	2018	India
SRR31645823	B19724	2018	India
SRR31645821	B19725	2018	India
SRR31645820	B19726	2018	India
SRR31645819	B19727	2018	India
SRR31645818	B19728	2018	India
SRR31645817	B19730	2018	India
SRR31645816	B19731	2018	India
SRR31645815	B19736	2018	India
SRR31645814	B19737	2018	India
SRR31645813	B19738	2018	India
SRR31645812	B19739	2018	India
SRR31645810	B19740	2018	India
SRR31645809	B19741	2018	India
SRR31645808	B19742	2018	India
SRR31645807	B19743	2018	India
SRR31645806	B19745	2018	India
SRR31645805	B19746	2018	India
SRR31645804	B19747	2018	India
SRR31645803	B19748	2018	India
SRR31645802	B19749	2018	India
SRR31645801	B19750	2018	India
SRR31646023	B19751	2018	India
SRR31646022	B19752	2018	India
SRR31646021	B19753	2018	India
SRR31646020	B19754	2018	India
SRR31646019	B19755	2018	India
SRR31646018	B19756	2018	India
SRR31646017	B19758	2018	India
SRR31646016	B19759	2019	India
SRR31646015	B19760	2019	India
SRR31646014	B19761	2019	India
SRR31646012	B19762	2019	India
SRR31646011	B19763	2019	India
SRR31646010	B19766	2019	India
SRR31646009	B19767	2019	India
SRR31646008	B19768	2019	India
SRR31646007	B19769	2019	India
SRR31646006	B19770	2019	India
SRR31646005	B19771	2019	India
SRR31646004	B19772	2019	India
SRR31646003	B19773	2019	India
SRR31646001	B19774	2019	India
SRR31646000	B19775	2019	India
SRR31645999	B19776	2019	India
SRR31645998	B19777	2019	India
SRR31645997	B19778	2019	India
SRR31645996	B19779	2019	India
SRR31645995	B19780	2019	India
SRR31645994	B19781	2019	India
SRR31645993	B19782	2019	India
SRR31645992	B19783	2019	India
SRR31645990	B19785	2019	India
SRR31645989	B19786	2019	India
SRR31645988	B19787	2019	India
SRR31645987	B19788	2019	India
SRR31645986	B19789	2019	India
SRR31645985	B19790	2019	India

Sample accession no.	Strain	Year of isolation	Geographic origin
SRR31645984	B19792	2019	India
SRR31645983	B19793	2019	India
SRR31645982	B19794	2019	India
SRR31645981	B19795	2019	India
SRR31645979	B19797	2019	India
SRR31645978	B19798	2019	India
SRR31645977	B19799	2019	India
SRR31645976	B19800	2019	India
SRR31645975	B19805	2019	India
SRR31645974	B19807	2019	India
SRR31645973	B19880	2017	India
SRR31645972	B19884	2017	India
SRR31645971	B19885	2017	India
SRR31645970	B25971	2016	Germany
SRR31645968	B25972	2017	Germany
SRR31645967	B25973	2018	Germany
SRR31645966	B25974	2018	Germany
SRR31645965	B25975	2018	Germany
SRR31645964	B25976	2018	Germany
SRR31645963	B25977	2018	Germany
SRR31645962	B25978	2018	Germany
SRR31645961	B25979	2019	Germany
SRR31645960	B25980	2019	Germany
SRR31645959	B25981	2019	Germany
SRR31645893	B25982	2019	Germany
SRR31645892	B25983	2019	Germany
SRR31645891	B25984	2019	Germany
SRR31645890	B25985	2019	Germany
SRR31645889	B25986	2019	Germany
SRR31645888	B25987	2019	Germany
SRR31645887	B25988	2019	Germany
SRR31645886	B25989	2019	Germany
SRR31645885	B25990	2019	Germany
SRR31645884	B25991	2019	Germany
SRR31645882	B25992	2019	Germany
SRR31645881	B25993	2019	Germany
SRR31645880	B25994	2019	Germany
SRR31645879	B25995	2020	Germany
SRR31645878	B25996	2020	Germany
SRR31645877	B25997	2020	Germany
SRR31645876	B25998	2020	Germany
SRR31645875	B25999	2020	Germany
SRR31645874	B26000	2020	Germany
SRR31645873	B26001	2020	Germany
SRR31645871	B26002	2020	Germany
SRR31645870	B26003	2020	Germany
SRR31645869	B26004	2020	Germany
SRR31645868	B26005	2020	Germany
SRR31645867	B26007	2020	Germany
SRR31645866	B26008	2020	Germany
SRR31645865	B26009	2020	Germany
SRR31645864	B26010	2020	Germany
SRR31645863	B26011	2020	Germany
SRR31645800	B26012	2020	Germany
SRR31645798	B26014	2021	Germany
SRR31645797	B26015	2021	Germany
SRR31645796	B26016	2021	Germany
SRR31645795	B26017	2021	Germany
SRR31645794	B26018	2021	Germany
SRR31645793	B26019	2021	Germany
SRR31645792	B26020	2021	Germany
SRR31645791	B26021	2021	Germany
SRR31645790	B26022	2022	Germany
SRR31645789	B26023	2022	Germany
SRR31645786	B26024	2022	Germany
SRR31645785	B26025	2020	Germany
SRR31645784	B26026	2022	Germany
SRR31645783	B26027	2022	Germany
SRR31645782	B26028	2022	Germany

Sample accession no.	Strain	Year of isolation	Geographic origin
SRR31645781	B26029	2022	Germany
SRR31645780	B26030	2022	Germany
SRR31645779	B26031	2022	Germany
SRR31645778	B26032	2022	Germany
SRR31645777	B26033	2022	Germany
SRR31645775	B26034	2022	Germany
SRR31645774	B26035	2022	Germany
SRR31645773	B26036	2022	Germany
SRR31645772	B26037	2022	Germany
SRR31645771	B26038	2022	Germany
SRR31645770	B26039	2022	Germany
SRR31645769	B26040	2022	Germany
SRR31645768	B26041	2022	Germany
SRR31645767	B26042	2022	Germany
SRR31645766	B26043	2023	Germany
SRR31645764	B26044	2023	Germany
SRR31645763	B26045	2023	Germany
SRR31645762	B26046	2021	Austria
SRR31645761	B26047	2022	Austria
SRR31645760	B26048	2019	Switzerland
SRR31645759	B26049	2017	Cambodia
SRR31645758	B26050	2019	Finland
SRR31645757	B26051	2019	Finland
SRR31645756	B26052	2019	Finland
SRR31645755	B26053	2019	Finland
SRR31645753	B26054	2019	Finland
SRR31645752	B26055	2020	Finland
SRR31645751	B26056	2020	Finland
SRR31645750	B26057	2020	Finland
SRR31645749	B26058	2021	Finland
SRR31645748	B26059	2021	Finland
SRR31645747	B26060	2021	Finland
SRR31645746	B26061	2019	Estonia
SRR31645745	B26062	2019	Poland
SRR31645744	B26063	2019	Poland
SRR31645742	B26064	2019	Poland
SRR31645741	B26065	2019	Poland
SRR31645740	B26066	2019	Poland
SRR31645739	B26067	2019	Poland
SRR31645738	B26068	2019	Iraq
SRR31645737	B26069	2019	Iraq
SRR31645736	B26070	2019	Iraq
SRR31645735	B26071	2019	Iraq
SRR31645734	B26072	2019	Iraq
SRR31645733	B26073	2019	Iraq
SRR31645731	B26074	2019	Iraq
SRR31645730	B26075	2019	Iraq
SRR31645729	B26076	2019	Iraq
SRR31645728	B26077	2019	Iraq
SRR31645727	B26078	2019	Iraq
SRR31645726	B26079	2019	Iraq
SRR31645725	B26080	2019	Iraq
SRR31645724	B26081	2019	Iraq
SRR31645723	B26082	2019	Iraq
SRR31645722	B26083	2020	Iraq
SRR31645720	B26084	2020	Iraq
SRR31645719	B26085	2020	Iraq
SRR31645718	B26086	2020	United Arab Emirates
SRR31645717	B26087	2020	United Arab Emirates
SRR31645716	B26088	2020	United Arab Emirates
SRR31645715	B26089	2020	United Arab Emirates
SRR31645714	B26090	2020	United Arab Emirates
SRR31645713	B26091	2020	United Arab Emirates
SRR31645712	B26092	2020	United Arab Emirates
SRR31645711	B26093	2020	United Arab Emirates
SRR31645709	B26096	2020	United Arab Emirates
SRR31645708	B26097	2020	United Arab Emirates
SRR31645707	B26098	2020	United Arab Emirates
SRR31645706	B26099	2020	United Arab Emirates

Sample accession no.	Strain	Year of isolation	Geographic origin
SRR31645705	B26100	2020	United Arab Emirates
SRR31645958	B26101	2020	United Arab Emirates
SRR31645957	B26102	2020	United Arab Emirates
SRR31645956	B26103	2020	United Arab Emirates
SRR31645955	B26104	2020	United Arab Emirates
SRR31645954	B26105	2020	United Arab Emirates
SRR31645952	B26106	2020	United Arab Emirates
SRR31645951	B26107	2020	United Arab Emirates
SRR31645950	B26108	2020	United Arab Emirates
SRR31645949	B26109	2020	United Arab Emirates
SRR31645948	B26110	2020	United Arab Emirates
SRR31645947	B26111	2020	United Arab Emirates
SRR31645946	B26112	2020	United Arab Emirates
SRR31645945	B26113	2020	United Arab Emirates
SRR31645944	B26114	2022	Bangladesh
SRR31645943	B26115	2022	Bangladesh
SRR31645941	B26116	2022	Bangladesh
SRR31645940	B26117	2022	Bangladesh
SRR31645939	B26118	2022	Bangladesh
SRR31645938	B26119	2022	Bangladesh
SRR31645937	B26120	2022	Bangladesh
SRR31645936	B26121	2022	Bangladesh
SRR31645935	B26122	2022	Bangladesh
SRR31645934	B26123	2022	Bangladesh
SRR31645933	B26124	2022	Bangladesh
SRR31645932	B26125	2022	Bangladesh
SRR31645929	B26126	2022	Bangladesh
SRR31645928	B26127	2022	Bangladesh
SRR31645927	B26129	2022	Bangladesh
SRR31645704	B26130	2022	Bangladesh
SRR31645703	B26131	2022	Bangladesh
SRR31645702	B26132	2022	Bangladesh
SRR31645701	B26133	2022	Bangladesh
SRR31645700	B26134	2022	Bangladesh
SRR31645699	B26135	2022	Bangladesh
SRR31645698	B26136	2022	Bangladesh
SRR31645696	B26137	2022	Bangladesh
SRR31645695	B26138	2022	Bangladesh
SRR31645694	B26140	2022	Bangladesh
SRR31645693	B26141	2022	Bangladesh
SRR31645692	B26142	2022	Bangladesh
SRR31645691	B26143	2022	Bangladesh
SRR31646044	B27812	2017	USA
SRR31646043	B27813	2021	USA
SRR31646042	B27814	2021	USA
SRR31646041	B27815	2022	USA
SRR31646039	B27816	2021	Canada
SRR31646038	B27817	2021	USA
SRR31646037	B27818	2021	USA
SRR31646036	B27819	2022	USA
SRR31646035	B27820	2020	USA
SRR31646034	B27821	2020	USA
SRR31646033	B27822	2022	USA
SRR31646032	B27823	2023	USA
SRR31646031	B27824	2023	USA
SRR31646030	B27825	2023	Canada
SRR31646028	B27826	2023	USA
SRR31646027	B27827	2023	USA
SRR31645926	B27828	2023	USA
SRR31645925	B27829	2023	USA
SRR31645924	B27830	2023	USA
SRR26858049	SRA725	2023	Singapore
SRR27198731	SAMN38471362	2023	USA
SRR27198732	SAMN38471361	2023	USA
SRR27198733	SAMN38471360	2023	USA
SRR27198734	SAMN38471359	2023	USA
SRR27198735	SAMN38471358	2023	USA
SRR27198736	SAMN38471357	2022	USA
SRR27198737	SAMN38471356	2023	USA

Sample accession no.	Strain	Year of isolation	Geographic origin
SRR27198738	SAMN38471355	2022	USA
SRR27198739	SAMN38471363	2023	USA
SRR27198740	SAMN38471354	2022	USA
SRR27198741	SAMN38471353	2022	USA

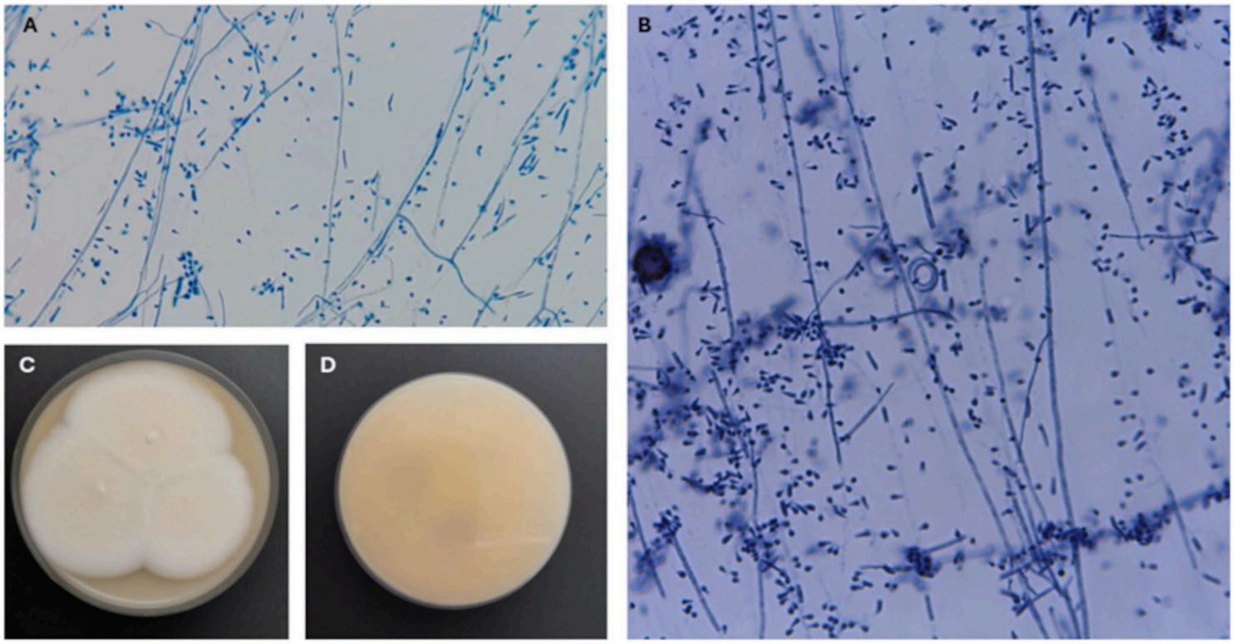

Appendix Figure. Morphologic characterization of the *Trichophyton indotineae* isolate. A, B) Microscopic features in a lactophenol cotton blue preparation: developing macroconidia, sessile microconidia, spiral hyphae, and released microconidia. C, D) Colony characteristics (Lactrimel, 2 weeks, 25°C).
